# Supplementary material for: A Million-Cow Genome-Wide Association Study of Three Fertility Traits in U.S. Holstein Cows
Source: Int J Mol Sci. 2023 Jun 22;24(13):10496. doi: 10.3390/ijms241310496 (PMC10341978; doi:10.3390/ijms241310496)

Supplemental Materials

A million-cow genome-wide association study of three fertility traits in U.S. Holstein cows

Zuoxiang Liang, Dzianis Prakapenka, Paul M. VanRaden, Jicai Jiang, Li Ma and Yang Da

Table S1. Top 1000 significant additive effects of DPR. (Excel)

Table S2. Significant dominance effects of DPR. (Excel)

Table S3. Top 1000 significant additive effects of CCR. (Excel)

Table S4. Significant dominance effects of CCR. (Excel)

Table S5. All 726 significant additive effects of HCR. (Excel)

Table S6. Significant dominance effects of HCR. (Excel)

Table S7. Common SNP additive effects of DPR, CCR and HCR. (Excel)

Table S8. Average phenotypic values and negative impact of recessive genotypes of thirteen SNPs. (Excel)

Table S9. Gene Ontology biological processes of candidate genes with additive effects for DPR. (Excel)

Table S10. Gene Ontology biological processes of candidate genes with additive effects for HCR. (Excel)

Table S11. SNP chips and density in this study. (Excel)

Table S12. Basic statistics of phenotypic residuals of three fertility traits after removing fixed nongenetic effects from the 12/2022 U.S. Holstein genomic evaluation.

Trait	Number of cows	Mean	Minimum value	Maximum value	Standard deviation
DPR	1,194,736	8.31	-54	75.07	25.31
CCR	1,001,374	12.53	-117.27	97.35	34.79
HCR	1,152,219	15.36	-107.18	111.24	36.66

DPR is daughter pregnancy rate. CCR is cow conception rate. HCR is heifer conception rate. The phenotypic observations of these traits were in percentages.

Figure S1.

Summary of GO biological process, cellular component, and molecular function for candidate genes of additive effects of DPR in Table 1.

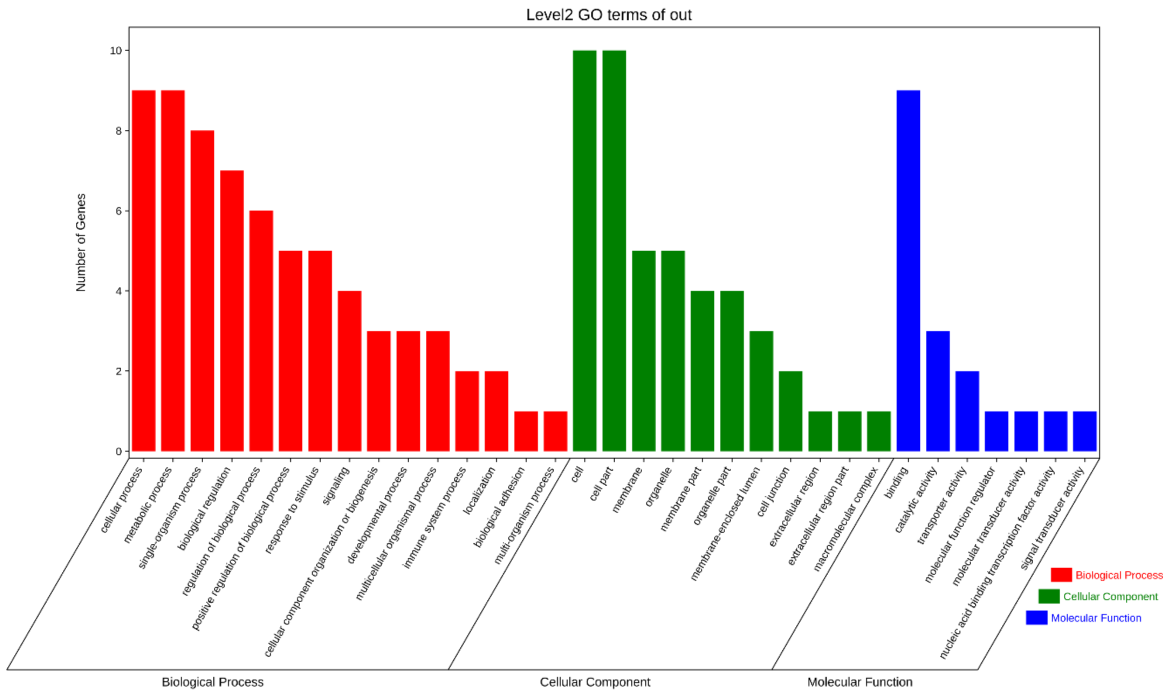

Figure S2.

Summary of GO biological process, cellular component, and molecular function for candidate genes of additive effects of HCR in Table 3.

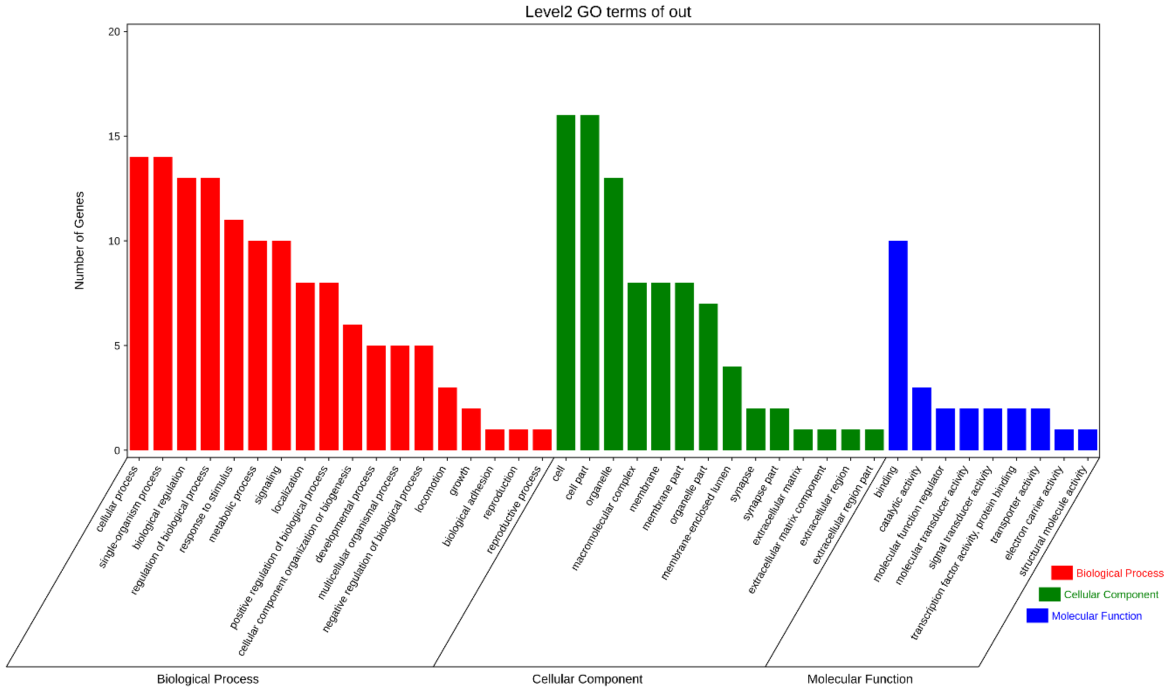

Supplement: Supplementary file 1 [file ijms-24-10496-s001.zip › Supplementary_materials/Supplementary_material.pdf]
